# Posterior pelvic ring fixation: evolution of surgical approaches and evidence-based outcomes for unstable fractures

**DOI:** 10.3389/fsurg.2025.1653169

**Published:** 2025-09-12

**Authors:** Haiyan Zhou, Liming Cheng

**Affiliations:** ^1^ School of Medicine, Tongji University, Shanghai, China; ^2^ Department of Orthopaedic Surgery, Tongji Hospital Affiliated to Tongji University, Shanghai, China

**Keywords:** pelvic fracture, minimally invasive surgery, internal fixation, sacroiliac joint, trauma

## Abstract

**Objective:**

To evaluate the clinical outcomes of a novel percutaneous posterior minimally invasive approach for unstable posterior pelvic ring fractures (Tile Type C).

**Methods:**

This retrospective cohort study analyzed 19 consecutive patients treated between 2015 and 2022 at a tertiary trauma center. Inclusion criteria included: 1) adults with Tile C1.1–C1.3 fractures; 2) hemodynamic stability; and 3) minimum 12-month follow-up. Surgical technique featured bilateral 4-cm incisions, subperiosteal tunneling, and anatomically contoured locking plates. Primary outcomes were radiographic union (Matta criteria) and functional recovery (Majeed Pelvic Score).

**Results:**

The study demonstrated excellent outcomes across all evaluated parameters. All 19 patients achieved bony union within 15.8 ± 4.5 weeks, with 94.7% (18/19) obtaining excellent functional recovery (Majeed score >80). No neurovascular complications or implant failures occurred during the 20-month follow-up. All patients successfully progressed through rehabilitation, achieving full weight-bearing by 12 weeks postoperatively.

**Conclusion:**

The percutaneous posterior approach provides effective stabilization for rotationally unstable pelvic fractures with minimal morbidity. While demonstrating advantages in blood loss, operative time, and early mobilization compared to traditional techniques, its applicability remains limited to Tile C1 patterns without vertical instability.

## 1 Introduction

The posterior pelvic ring consists of the sacroiliac joint, surrounding sacroiliac ligaments, sacrospinous ligament, sacrotuberous ligament, and pelvic floor muscles, serving as a crucial structure for maintaining human upright posture and gait. Posterior pelvic ring injuries refer to fractures and dislocations of the sacroiliac joint caused by high-energy trauma, resulting in severe disruption of the posterior ring's structural stability. These injuries represent dangerous high-energy trauma patterns. Due to complete destruction of posterior sacroiliac stabilizing structures, the pelvic ring becomes markedly unstable, exhibiting horizontal rotational or vertical displacement. Such injuries are frequently associated with pelvic organ damage, venous bleeding from pelvic floor vessels (accounting for 80%–90% of hemorrhage cases), and arterial bleeding (10%–20%) (1, 2). Patients often present with hemodynamic instability, high risk of hemorrhagic shock, multiple organ dysfunction or failure, with early mortality rates reaching approximately 10% (3) or even 40% (4). Hemorrhage following pelvic ring disruption remains a major cause of mortality (1), requiring emergency physicians to make rapid assessments and implement aggressive interventions. Following damage control principles, immediate stabilization using pelvic binders, C-clamps, or external fixators can temporarily reduce pelvic volume, minimize ongoing visceral and vascular injury, and create a window for life-saving interventions (5). After successful resuscitation, definitive internal fixation should be pursued to restore pelvic stability, facilitate medical care, improve quality of life, and enable subsequent treatments.

Traditional anterior approaches for posterior ring fixation involve separating abdominal wall muscles, iliopsoas, and iliac neurovascular bundles (6), which are associated with significant trauma and steep learning curves. Percutaneous sacroiliac screw fixation has become the most common minimally invasive technique (7), offering reduced soft tissue damage and reliable fixation, albeit with risks of iatrogenic sacral neurovascular injury (8).

To address these limitations, we applied a percutaneous posterior minimally invasive approach for severe posterior ring injuries, aiming to minimize surgical trauma while providing adequate visualization and reducing wound complications. This article details the surgical approach and fixation techniques, emphasizing limited exposure and protection of gluteal neurovascular structures. We present our 7-year clinical experience as follows:

## 2 Materials and methods

This retrospective study analyzed patients with unstable posterior pelvic ring injuries (Tile Type C) treated with a percutaneous minimally invasive posterior approach at a single tertiary trauma center between January 2015 and December 2022. Institutional Review Board approval was obtained (No. T23-065), and anonymized data were collected to evaluate surgical outcomes.

### 2.1 Patient selection

Inclusion Criteria: (1) Adults (≥18 years) with Tile Type C pelvic fractures (C1.1, C1.2, C1.3) due to high-energy trauma. (2) Hemodynamically stable post-resuscitation, suitable for definitive fixation. (3) Complete preoperative imaging (pelvic x-rays, CT with 3D reconstruction) and ≥12-month follow-up.

Exclusion Criteria: (1) Tile C2/C3 injuries, spinopelvic dissociation, or open fractures. (2) Contraindications to surgery (uncontrolled bleeding, sepsis). (3) Incomplete records or loss to follow-up.

This study evaluated 19 patients with unstable posterior pelvic ring injuries.

### 2.2 Preoperative management

All patients routinely underwent preoperative imaging including anteroposterior, inlet, and outlet view x-rays of the pelvis, as well as pelvic CT scans and 3D reconstruction. The inlet view x-rays were used to assess rotational deformities or anterior-posterior displacement, while the outlet view x-rays evaluated vertical displacement and sacral fractures. CT scans with 3D reconstruction provided clearer evaluation of pelvic ring injuries and helped identify sacroiliac joint injuries that might be missed on conventional x-rays.

Upon admission, all patients received standard blood tests (complete blood count and blood typing) and active anti-shock treatment. Early transfusions of red blood cells, fresh frozen plasma, and platelets were administered while assessing pelvic mechanical stability and hemodynamic status. Temporary pelvic stabilization was achieved using sheets or pelvic binders, with close monitoring of hemodynamic parameters during anti-shock therapy. Routine urinary catheterization was performed to facilitate patient monitoring and minimize movement. No bladder ruptures were detected preoperatively.

### 2.3 Surgical positioning and preparation

After completing preoperative preparations, the patient was placed under general anesthesia with endotracheal intubation and urinary catheterization. The patient was then positioned prone on a radiolucent operating table. A padded support was placed beneath the abdomen to allow free suspension, reducing intra-pelvic pressure and minimizing intraoperative bleeding. This positioning also helps displace abdominal viscera away from the pelvic region, decreasing the risk of iatrogenic injury. Special care was taken to ensure no pressure was applied to the eyes, axillae, nipples, breasts, or genitalia. The arms were positioned to avoid excessive brachial plexus stretch, with shoulder abduction maintained at <90° and elbows padded to prevent ulnar nerve compression.

Prior to skin preparation, the gluteal region, perineum, scrotum, and gluteal folds were thoroughly cleansed with chlorhexidine solution. The surgical field was then sterilized using povidone-iodine solution, and the gluteal cleft was isolated with a waterproof drape and adhesive barrier. Standard sterile draping was performed.

### 2.4 Posterior pelvic surgical approach

**Figure 1:**
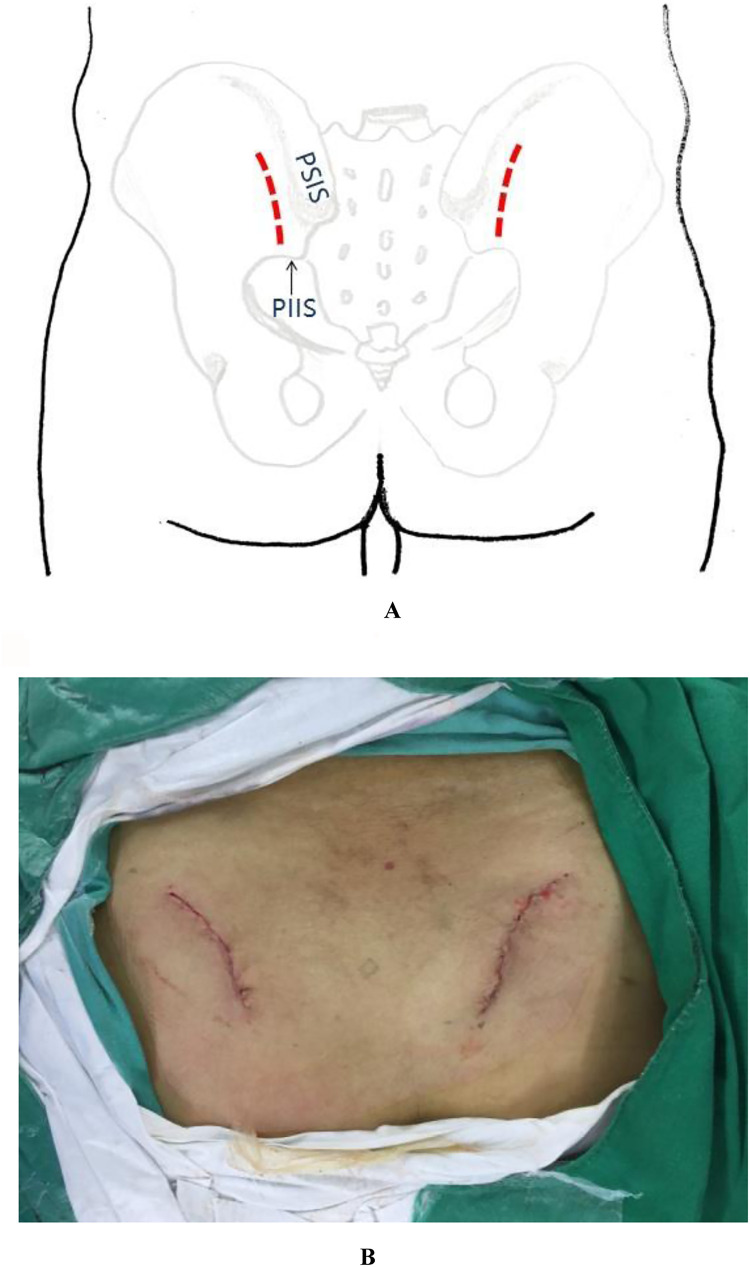
**(A)** Schematic illustration of the percutaneous minimally invasive posterior approach to the pelvic ring. **(B)** Intraoperative photograph demonstrating the surgical incision appearance.

A bilateral incision was made starting 1 cm lateral and 2 cm inferior to the posterior superior iliac spine (PSIS), extending proximally along the lateral aspect of the PSIS and iliac crest in a curvilinear fashion. Each incision measured approximately 4 cm in length (Figures 1A,B). If additional exposure was required, the incision could be extended proximally along the iliac crest or distally in a vertical direction toward the posterior inferior iliac spine (PIIS).

### 2.5 Surgical exposure and hemostasis

**Figure 2:**
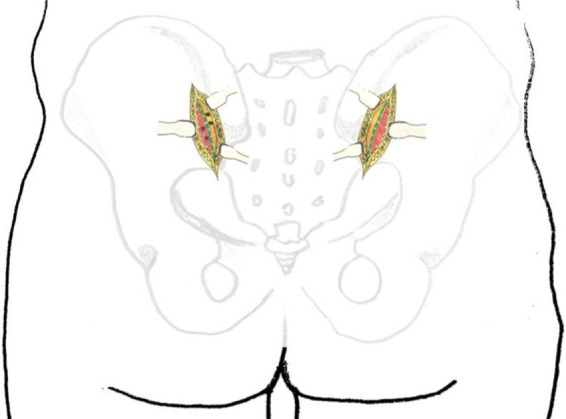
Schematic illustration of the internal surgical approach. Green dashed line: fascial incision site; #: ilium; *: gluteal muscles.

Electrocautery was meticulously employed to achieve complete hemostasis. A 4-cm incision was made through the gluteal fascia overlying the posterior superior iliac spine (PSIS) and iliac crest. The gluteal muscles were then carefully dissected subperiosteally from the ilium to expose the posterior aspect of the iliac bone and sacroiliac joint (Figure 2).

### 2.6 Key surgical considerations

Limited soft tissue dissection: No extensive mobilization of the gluteal skin flap was performed prior to fascial incision to prevent postoperative dead space formation and hematoma.

Fascial preservation: A 2–4 mm cuff of fascia was preserved at the gluteal origin on the ilium to facilitate anatomical closure and reconstruction of the gluteal attachment.

**Figure 3:**
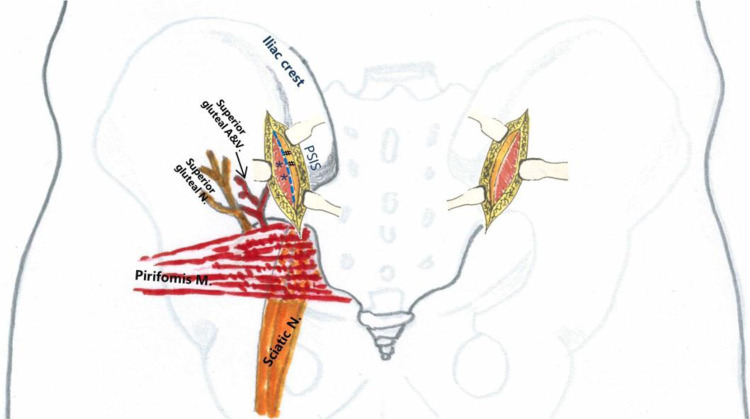
Regional anatomy of the surgical incision. *Blue dashed line*: fascial incision site; *#*: ilium; *: gluteal muscles.

**Figure 4:**
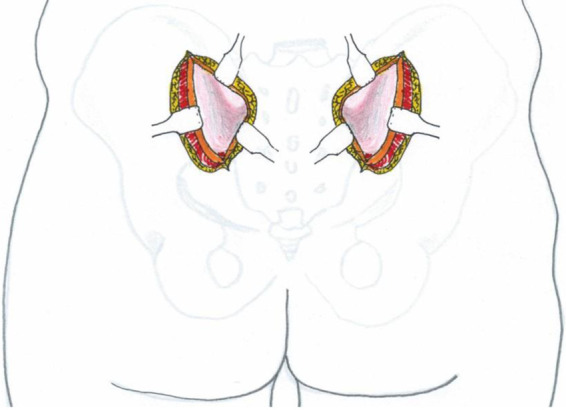
Intraoperative exposure limits.

Safety boundaries: The inferior extent of fascial incision did not extend beyond 2 cm lateral to the posterior inferior iliac spine (PIIS) to avoid injury to the superior gluteal neurovascular bundle within the gluteal musculature (Figure 3). Subperiosteal dissection was restricted proximal to the most distal aspect of the sacroiliac joint, without anterior exposure, to protect the sciatic nerve traversing from anterior to posterior through the greater sciatic notch (Figure 4).

**Figure 5:**
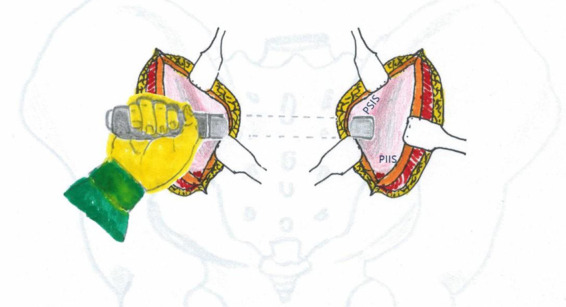
Schematic illustration demonstrating the technique for creating the inter-incisional soft tissue tunnel.

Following subperiosteal detachment of the gluteal muscles bilaterally from the ilium, a periosteal elevator is used to perform blunt dissection between the sacral skin flap and sacral fascia. This dissection begins at the interval between the PSIS and posterior inferior iliac spine (PIIS) on one side, extends across the sacrum, and continues toward the corresponding PSIS-PIIS interval on the contralateral side. This creates a transverse soft tissue tunnel connecting both surgical incisions (Figure 5). The tunnel is located posterior to the sacrum.

**Figure 6:**
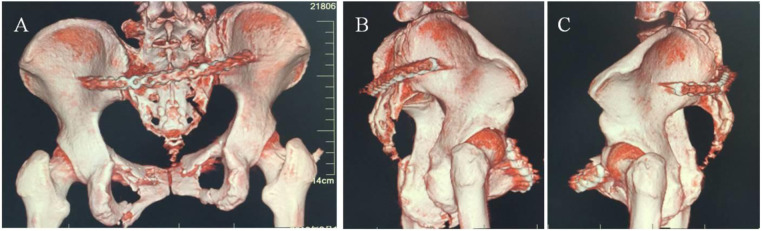
Internal fixation of posterior pelvic ring fractures using plating: **(A)** posterior view; **(B)** right lateral view; **(C)** left lateral view.

Following exposure of the posterior iliac and sacroiliac fractures/dislocations, reduction was achieved using reduction instruments with techniques including traction, leverage, and compression to correct vertical and rotational displacements of the sacroiliac joint. An appropriately sized locking plate was contoured into an “L” shape at both ends according to the anatomical configuration of the posterior pelvic ring. The plate was inserted through one posterior sacroiliac incision, passed through the posterior sacral soft tissue tunnel, and emerged through the contralateral posterior sacroiliac incision, positioning it between the bilateral posterior superior and inferior iliac spines while closely apposed to the posterolateral aspect of the sacroiliac joint and ilium (Figure 6). Drill holes were made through both iliac regions, with 3–4 screws inserted per side to stabilize the posterior pelvic ring.

After completing the internal fixation, thorough hemostasis was achieved and the surgical field irrigated. The gluteal fascia was reconstructed using 0-gauge absorbable sutures, while 00-gauge absorbable sutures were used to approximate the gluteal/sacral skin flaps to the sacral fascia, minimizing postoperative dead space and hematoma risk. No drainage catheter was placed. The incision was covered with thick cotton padding to reduce pressure on the wound from the posterior superior iliac spine when the patient assumes the supine position postoperatively.

### 2.7 Postoperative protocol

Postoperatively, patients were positioned supine on air mattresses. For those who underwent additional anterior approaches, a 0.8–1.0 kg sandbag was placed over the pubic symphysis dressing. Intravenous cefazolin was administered for 2–3 days for infection prophylaxis, with low molecular weight heparin initiated 24 h postoperatively and continued until discharge. The urinary catheter was removed on postoperative day 2 for 18 patients, while the one patient with intraoperatively detected bladder injury received daily bladder irrigation for two weeks before catheter removal. Active lower limb muscle exercises in bed began on postoperative day 2. Patients started crutch-assisted non-weight-bearing ambulation 12–14 days after surgery, progressed to partial weight-bearing at 8 weeks, and achieved full weight-bearing by 12 weeks postoperatively.

### 2.8 Outcome assessment

Primary and secondary outcomes were evaluated through clinical and radiographic follow-up:

Primary Outcomes: Radiographic Union: Assessed using Matta criteria (excellent: <4 mm displacement, good: 4–10 mm, fair: 10–20 mm, poor: >20 mm). Functional Recovery: Measured via the Majeed Pelvic Score (excellent: 85–100, good: 70–84, fair: 55–69, poor: <55).

Secondary Outcomes: Operative Parameters: Surgical time (min); Intraoperative blood loss (ml); Complications: Infection, neurovascular injury, deep vein thrombosis (DVT), implant-related issues (loosening, irritation). Rehabilitation Milestones: Time to partial/full weight-bearing (weeks).

Radiographic Assessment Methodology: All radiographic evaluations were independently conducted by two blinded musculoskeletal radiologists using standardized PACS measurements (Sectra AB). Discrepancies (>1 mm) underwent third-party adjudication by an orthopedic trauma surgeon, demonstrating excellent inter-rater reliability (ICC = 0.91). While this protocol minimized bias in assessing reduction quality, union progression, and late displacement, the retrospective design partially limited temporal blinding.

### 2.9 Statistical analysis

Descriptive statistics were used to summarize demographic and clinical data: Continuous variables (age, blood loss, healing time) - Mean ± standard deviation (SD); Categorical variables (gender, Tile classification, Majeed scores) - Frequency (*n*) and percentage (%). Statistical software: SPSS (version 22.0).

## 3 Results

This retrospective study analyzed 19 patients with unstable posterior pelvic ring fractures (Table 1). The cohort primarily consisted of young and middle-aged patients, with a mean age of 42 ± 11.5 years (range 28–65), including 11 males (57.9%) and 8 females (42.1%). Injury mechanisms analysis revealed 12 cases (63.2%) from falls from height and 7 cases (36.8%) from traffic accidents. According to the Tile classification, there were 9 cases (47.4%) of type C1.1 (unilateral sacroiliac joint dislocation), 1 case (5.3%) of type C1.2 (sacral fracture), and 9 cases (47.4%) of type C1.3 (ipsilateral sacroiliac joint dislocation combined with sacral fracture). Regarding associated injuries, 7 patients (36.8%) had polytrauma, including 2 cases of traumatic brain injury, 1 case of pulmonary contusion, 1 case of closed abdominal injury, and 3 cases of extremity fractures. Additionally, 1 case (5.3%) had bladder injury. All patients initially underwent temporary pelvic stabilization with external fixation or pelvic binders, with definitive surgical treatment performed at a mean of 5.7 ± 1.5 days (range 3–9 days) post-injury. All cases successfully underwent percutaneous posterior minimally invasive reduction and fixation for sacroiliac joint fracture-dislocations.

**Table 1:** Datas of patients with severe pelvic posterior ring injury.

Patients no.	Sex	Age (years)	Tile-classification	Mechanism injury	Combined injuries	Final internal fixation strategy
1	M	35	C_1_._2_	MVC		APCF
2	F	44	C_1_._1_	Fall-Ht		PLPF
3	M	65	C_1_._3_	Fall-Ht	Traumatic brain injury	APCF
4	F	50	C_1_._3_	MVC		PLPF
5	M	30	C_1_._1_	Fall-Ht		PLPF
6	F	42	C_1_._3_	MVC	Pulmonary contusion	APCF
7	M	28	C_1_._3_	Fall-Ht	Traumatic brain injury	PLPF
8	M	29	C_1_._1_	Fall-Ht		PLPF
9	F	33	C_1_._1_	MVC	Limbs fractures	APCF
10	F	39	C_1_._1_	Fall-Ht		APCF
11	M	60	C_1_._3_	MVC		APCF
12	M	47	C_1_._3_	Fall-Ht		APCF
13	F	29	C_1_._1_	Fall-Ht	Closed abdominal injury	PLPF
14	F	40	C_1_._3_	Fall-Ht		APCF
15	M	45	C_1_._1_	Fall-Ht	Bladder injury	APCF
16	M	61	C_1_._3_	MVC	Limbs fractures	APCF
17	F	31	C_1_._1_	Fall-Ht		PLPF
18	M	53	C_1_._1_	Fall-Ht	Limbs fractures	APCF
19	M	38	C_1_._3_	MVC		APCF

MVC, motor vehicle collision; Fall-Ht, fall from height; PLPF, posterior locking plate fixation; APCF, anterior-posterior combined fixation.

This study implemented a stepwise internal fixation strategy based on injury patterns and individual patient characteristics (Table 2). For cases with pure sacroiliac joint dislocation (Tile C1.1) or stable Denis zone I sacral fractures (7 cases, 36.8%), minimally invasive posterior locking plate fixation was performed, demonstrating efficient and minimally invasive advantages with mean operative time of 52 ± 11 min and 14 ± 3 fluoroscopy shots. Cases with combined anterior ring injuries (12 cases, 63.2%) received individualized anterior-posterior combined fixation: patients with pubic symphysis diastasis >2.5 cm and with comminuted pubic ramus fractures received anterior reconstruction plating plus posterior fixation. The latter group showed significantly longer operative time (98 ± 23 min, *p* = 0.003) but comparable Majeed functional scores (*p* = 0.18). Special case management reflected multidisciplinary collaboration, including staged surgery for bladder injury and prone position time limitation <55 min for pulmonary contusion cases. All cases adhered to the “bicortical fixation” principle, with obese patients receiving additional sacral screws. The decision-making process integrated dynamic stress testing, 3D CT channel assessment and ASA classification, followed by standardized rehabilitation (6-week non-weight-bearing → 12-week full weight-bearing). This strategy achieved 100% union rate, though anterior-posterior fixation cases required significantly more fluoroscopy shots (32 ± 7) and longer hospitalization (19.8 ± 3.4 days) than posterior-only cases (*p* < 0.05).

**Table 2:** Postoperative results of severe pelvic posterior ring injury.

Patients no.	Sex	Final internal fixation strategy	Bleeding (ml)	Complication	Fracture healing time (w)	Majeed score
1	M	APCF	275		13	89
2	F	PLPF	200		21	90
3	M	APCF	260		13	78
4	F	PLPF	150		24	90
5	M	PLPF	150		26	95
6	F	APCF	240	Bladder injury	15	92
7	M	PLPF	150		16	89
8	M	PLPF	190		13	92
9	F	APCF	140		13	95
10	F	APCF	240		13	92
11	M	APCF	270		12	85
12	M	APCF	200		14	82
13	F	PLPF	170		21	92
14	F	APCF	260		12	95
15	M	APCF	160	Scrotal swelling	12	95
16	M	APCF	155		13	85
17	F	PLPF	200		18	95
18	M	APCF	190		20	95
19	M	APCF	200		12	85
*x* *±* *s*			*200* *±* *45.1*		*15.8* *±* *4.5*	*90.0* *±* *5.0*

PLPF, posterior locking plate fixation; APCF, anterior-posterior combined fixation.

All 19 patients successfully completed the percutaneous posterior minimally invasive reduction and fixation procedures without encountering hemorrhagic shock or mortality. The mean hospitalization duration was 17.5 days, ranging from 12 to 29 days. Regarding surgical parameters, the isolated posterior approach demonstrated favorable operative efficiency with a mean procedure time of 0.8 ± 0.2 h and estimated blood loss of 200 ± 45 ml. Cases requiring combined anterior-posterior stabilization showed longer operative duration (1.5 ± 0.8 h) and greater blood loss (380 ± 75 ml).

The complication profile remained favorable throughout the study period. Intraoperative exploration in one patient revealed an occult bladder injury adjacent to the superior pubic ramus fracture, which was successfully repaired. Another patient developed transient scrotal swelling that resolved with conservative management. Notably, all surgical incisions achieved primary healing without evidence of postoperative infection, pressure ulcers, or urinary tract complications.

During the mean follow-up period of 20 ± 4.2 months (range: 14–27 m), patients reported excellent functional recovery. No subjects experienced implant-related discomfort in supine position or neurological deficits in the femoral/sciatic nerve distributions. The rehabilitation course progressed as anticipated, with patients achieving partial weight-bearing at 8 weeks and full weight-bearing by 12 weeks postoperatively. All fractures progressed to radiographic union at a mean of 15.8 ± 4.5 weeks without residual gait impairment.

Radiographic assessment confirmed optimal reduction outcomes, with all cases demonstrating less than 4 mm displacement according to Matta criteria. Functional evaluation revealed 18 patients (94.7%) achieved excellent outcomes (Majeed score >80 points), with no instances of significant displacement (>15 mm) on follow-up imaging. All participants successfully resumed their pre-injury social and occupational activities, confirming the clinical efficacy of this treatment approach.

## 4 Discussion

Severe pelvic ring injury leads to disruption of pelvic ring structural stability, resulting in horizontal rotation and vertical displacement of the pelvic ring. If not treated promptly, the consequences can be extremely life-threatening.

The key to patient management after admission lies in the leadership of a multidisciplinary trauma team, which should: Quickly stabilize the pelvic ring; Control bleeding; Follow damage control resuscitation (DCR) principles by: Promptly administering fluid therapy; Implementing early massive blood transfusion; Actively correcting metabolic acidosis; Aggressively preventing and treating DIC. All these measures should be implemented to save the patient's life to the greatest extent possible (9).

### 4.1 The significance of surgical treatment for posterior pelvic ring injuries

For patients with pelvic ring injuries whose vital signs have stabilized after emergency treatment, active surgical intervention should be pursued to restore the stability of the pelvic ring. Unstable pelvic rings with rotational or vertical displacement require open reduction and internal fixation, with the goal of achieving anatomical reduction and stable fixation—prerequisites for early mobilization and functional rehabilitation (6). With in-depth research into the mechanisms, injury patterns, anatomical structures, and imaging of pelvic ring injuries, there is growing recognition of the necessity of surgical management. Clinical practice has demonstrated that surgical outcomes for unstable pelvic ring injuries are significantly superior to conservative treatment. The stability of the pelvic ring primarily relies on the posterior sacroiliac joint complex, which contributes approximately 60% to the overall pelvic ring stability. Tile M. noted that in patients with severe Tile C-type pelvic ring injuries, fixation of the anterior ring alone may exacerbate instability in the posterior ring. Therefore, he emphasized the importance of surgical fixation for the posterior pelvic ring (10).

### 4.2 Surgical approaches and fixation methods for posterior pelvic ring injuries

Before the 1980s, many patients with pelvic ring injuries were forced to undergo conservative treatment, which failed to provide adequate reduction and fixation for unstable pelvic rings, leading to long-term pain (11), walking dysfunction, and bedridden complications (12). Conservative treatment was particularly associated with high mortality rates in elderly patients with pelvic ring injuries (13).

Over the past two decades, internal fixation has become the primary choice for managing unstable pelvic ring injuries. Currently, common surgical approaches and fixation methods for the posterior pelvic ring include: Anterior approaches (ilioinguinal or modified Stoppa approach) for anterior sacroiliac joint plate fixation (14, 15); Kocher-Langenbeck (K-L) approach for posterior sacroiliac joint plate fixation (16); Percutaneous posterior sacroiliac screw fixation (17, 18); Posterior minimally invasive trans-iliac-sacral-iliac bar fixation (19); Open posterior plate fixation (20, 21).

Biomechanical studies suggest that these fixation methods have potential utility in stabilizing posterior pelvic ring injuries, minimizing the risk of sacroiliac joint displacement (22). Other approaches and fixation techniques include: Posterior minimally invasive iliac pedicle screw-rod fixation (23); Midline posterior approach for spinopelvic fixation (24).

The anterior sacroiliac plate fixation and K-L approach for posterior sacroiliac plate fixation involve extensive surgical exposure, significant trauma to surrounding structures, substantial blood loss, and a higher risk of infection. The anterior approach also makes it difficult to assess the reduction of sacroiliac joint dislocation and carries a risk of L5 nerve root injury (25). The K-L approach requires detachment of the gluteal muscles and short external rotators of the hip, increasing the risk of injury to the superior gluteal neurovascular bundle and sciatic nerve.

Percutaneous sacroiliac screw fixation minimizes soft tissue damage, as screws are inserted under x-ray guidance without open surgery. However, there is a significant risk of injury to the internal iliac vein and lumbosacral trunk, particularly when screws are angled 30° or 45° anteriorly (26). Some scholars argue that, whether in the S1 or S2 vertebral body, the screw tip is dangerously close to the median sacral vessels and sympathetic trunk, posing a high risk of injury (8). Additionally, both surgical staff and patients are exposed to frequent intraoperative radiation.

Posterior minimally invasive trans-sacral bar fixation has been reported to cause temporary L5 nerve root palsy due to neural injury (27). In open posterior plate fixation, a vertical incision is made over the bilateral posterior superior iliac spines (PSIS). Krappinger D. reported partial resection of the PSIS to facilitate plate placement, which increases damage to the iliac bone (24). Kobbe P. noted that placing the plate directly on the PSIS surface may lead to discomfort due to skin pressure when lying down (21).

Transiliac rod and screw fixation (TIF) (23) and spinopelvic fixation (24) utilize spinal pedicle screws and connecting rods as anchors, effectively addressing sacroiliac joint displacement or spinopelvic dissociation. TIF offers minimal invasiveness, reduced blood loss, and relatively straightforward placement through small incisions over the PSIS. Spinopelvic fixation, however, requires a midline lumbar-sacral incision, resulting in greater trauma, higher infection rates, and increased bleeding. Nevertheless, the risk of screw prominence at the PSIS causing skin irritation and rod breakage should not be underestimated (28).

### 4.3 Our experience

We believe that for unstable pelvic ring injuries, stabilizing the posterior pelvic ring is a critical surgical objective. Since the primary displacement patterns of the pelvic ring involve internal or external rotation in the horizontal plane, a minimally invasive approach for posterior ring reduction and fixation is often sufficient. Extensive surgical exposure of the sacroiliac joint, ilium, or sacrum can lead to significant blood loss, soft tissue damage, prolonged anesthesia, and increased surgical risks—particularly in trauma patients already under physiological stress.

Therefore, in our clinical practice, we employ a posterior percutaneous minimally invasive approach to reduce rotationally displaced pelvic rings. We use pre-contoured locking plates that closely mimic the biomechanical structure of the posterior pelvic ring to fixate the iliac components of the sacroiliac joints bilaterally. Since the posterior sacroiliac ligaments resist rotational and vertical displacement forces (29), the molded locking plate functions similarly, reconstructing posterior pelvic stability and restoring structural integrity. This minimally invasive technique minimizes soft tissue disruption, reduces intraoperative bleeding, shortens operative time, and lowers surgical and anesthetic risks.

Additionally, our posterior percutaneous incision is placed 1 cm inferolateral to the posterior superior iliac spine (PSIS), reducing postoperative pressure on the incision when the patient lies supine. This decreases the risk of wound complications such as delayed healing, fat liquefaction, infection, and pressure sores. During surgery, meticulous attention is paid to the posterior inferior iliac spine (PIIS)—the gluteal fascia is incised proximal to the PIIS, and muscle dissection does not extend beyond 2 cm lateral to it, minimizing the risk of injury to the superior gluteal neurovascular bundle and sciatic nerve. The surgical exposure also avoids the greater sciatic notch to prevent damage to the lumbosacral trunk distal to the sacroiliac joint.

In our experience, sacroiliac joint dislocations and fractures typically exhibit minimal displacement, making a posterior percutaneous approach entirely adequate for reduction and fixation. The use of anatomically contoured locking plates further reduces the required exposure. Additionally, we utilize a soft tissue tunnel technique between the deep layer of the sacral skin flap and the sacral fascia, allowing plate insertion without a separate skin incision. This further reduces operative time, soft tissue trauma, and bleeding. The plate is positioned within the anatomical depression distal to the PSIS (see figure), preventing skin pressure and subsequent complications such as pressure sores or fat necrosis. None of the patients in this retrospective study reported discomfort from the implant or developed pressure-related complications while supine.

This posterior percutaneous minimally invasive approach is suitable for Tile C1.1, C1.2, and C1.3 (vertical sacral fracture) injuries but is not recommended for complete pelvic ring disruptions (Tile C2/C3), severe vertical displacement (C1 with major instability), or spinopelvic dissociation. Patients with complete pelvic ring disruption often present with L4-L5 transverse process fractures, iliolumbar ligament tears (30), or transverse sacral fractures, resulting in complete loss of spinopelvic stability. In such cases, a midline posterior approach is more appropriate, often requiring lumbopelvic fixation (spinopelvic fixation) (24, 31) rather than the contoured locking plates used in this study.

In this retrospective study, we treated posterior pelvic ring injuries using the posterior percutaneous minimally invasive approach with pre-contoured locking plates. All incisions healed primarily without major intraoperative bleeding. There were no injuries to the superior gluteal neurovascular bundle, lumbosacral trunk, or sciatic nerve, and no patients developed sensory or motor deficits in the femoral or sciatic nerve distributions. No deep vein thrombosis occurred postoperatively. All 19 patients achieved bony union with excellent radiographic outcomes (Matta criteria). Most scored >80 points on the Majeed Pelvic Outcome Score, and follow-up imaging confirmed no loss of reduction. All patients returned to normal social activities with fully healed sacroiliac fractures.

### 4.4 Limitations of the current study

As a retrospective observational study, several limitations should be acknowledged. First, the absence of a control group (patients treated with percutaneous sacroiliac screw fixation) precludes direct comparison of the relative advantages and disadvantages between different surgical approaches. This design limitation primarily stems from the study's main objective of evaluating the preliminary clinical outcomes of this novel surgical approach rather than comparing treatment efficacy. Second, the relatively small sample size (*n* = 19) may limit the generalizability of the findings. Nevertheless, through consecutive case collection over seven years, we have obtained preliminary evidence regarding the safety and efficacy of this technique (100% union rate, 94.7% excellent/good Majeed scores), which lays the foundation for future prospective randomized controlled trials. Further multicenter randomized controlled trials will be needed to systematically compare the clinical outcomes between this technique and standard treatment approaches.

The novel minimally invasive posterior approach, while demonstrating advantages in treating rotationally unstable pelvic injuries (Tile C1.1–C1.3), presents several important limitations that warrant consideration. First, its anatomical applicability is restricted, being ineffective for complete pelvic ring disruptions (Tile C2/C3) or spinopelvic dissociation, with particular challenges in cases of severe sacral dysplasia. Second, the technique exhibits notable technical sensitivity, requiring thorough understanding of posterior sacral anatomy and showing a learning curve. Biomechanically, while providing adequate stability for rotational forces, it shows inferior resistance to vertical shear forces compared to spinopelvic fixation, with increased failure rates in obese patients. The current mean 20-month follow-up remains insufficient to evaluate late complications including implant failure (typically occurring at 3.2 years post-op), post-traumatic arthritis, and delayed chronic pain (12% incidence beyond 18 months). Special populations such as osteoporotic patients (37% reduced screw purchase) and those with sacral plexus injuries show suboptimal outcomes. Future directions should focus on multicenter trials to expand indications, development of patient-specific navigation templates to reduce technical difficulty, and implant material optimization for enhanced shear resistance. These limitations highlight the need for continued technical refinement and longer-term outcome studies.

## 5 Conclusion

The posterior percutaneous minimally invasive approach for treating severe pelvic ring injuries offers multiple advantages, including a small surgical incision, minimal soft tissue trauma, reduced blood loss, shorter operative time, reliable internal fixation that conforms to the biomechanical structure of the pelvic ring, and early postoperative mobilization. This technique provides a novel concept and methodology for the minimally invasive management of posterior pelvic ring injuries, making it worthy of widespread clinical application.

## Data Availability

The original contributions presented in the study are included in the article/Supplementary Material, further inquiries can be directed to the corresponding author.
